# Association of Glutathione S-transferase gene polymorphism with bladder Cancer susceptibility

**DOI:** 10.1186/s12885-018-5014-1

**Published:** 2018-11-12

**Authors:** Tianbiao Zhou, Hong-Yan Li, Wei-Ji Xie, Zhiqing Zhong, Hongzhen Zhong, Zhi-Jun Lin

**Affiliations:** 1 Department of Nephrology, The Second Affiliated Hospital of Shantou University Medical College, Shantou, 515041 China; 2 Department of Nephrology, Huadu District People’s Hospital of Guangzhou, Southern Medical University, Guangzhou, China

**Keywords:** Bladder cancer, Gene polymorphism, GSTM1, GSTT1, GSTP1, Meta-analysis

## Abstract

**Background:**

We conducted a meta-analysis to evaluate the relationship between the glutathione S-transferase μ1 (GSTM1)– and glutathione S-transferase θ1 (GSTT1)– null genotypes and susceptibility to bladder cancer.

**Methods:**

We identified association reports from the databases of PubMed, Embase, the Cochrane Library and the China Biological Medicine Database (CBM disc) on July 1, 2017 and synthesized eligible investigations. Results were expressed using odds ratios (ORs) for dichotomous data, and we also calculated 95% confidence intervals (CIs).

**Results:**

In this meta-analysis, we found that the GSTM1-null genotype was associated with bladder cancer risk in the overall population, and individually in whites, Africans and Asians (overall population: OR = 1.40, 95% CI: 1.31–1.48, *P*<0.00001; whites: OR = 1.39, 95% CI: 1.26–1.54, *P*<0.00001; Africans: OR = 1.54, 95% CI: 1.16–2.05, *P* = 0.003; Asians: OR = 1.45, 95% CI: 1.33–1.59, *P*<0.00001). The GSTT1-null genotype was associated with bladder cancer risk in the overall population, but not in whites, in Africans or Asians (overall population: OR = 1.11, 95% CI: 1.01–1.22, *P* = 0.03; whites: OR = 1.16, 95% CI: 0.99–1.36, *P* = 0.07; Africans: OR = 1.07, 95% CI: 0.65–1.76, *P* = 0.79; Asians: OR = 1.05, 95% CI: 0.91–1.22, *P* = 0.51). Interestingly, a dual-null GSTM1–GSTT1 genotype was associated with bladder cancer risk in the overall population and in Asians (overall population: OR = 1.48, 95% CI: 1.15–1.92, *P* = 0.002; Asians: OR = 1.62, 95% CI: 1.15–2.28, *P* = 0.006). In conclusion, the GSTM1-null, GSTT1-null and dual-null GSTM1–GSTT1 genotypes might be associated with the onset of bladder cancer, but additional genetic-epidemiological studies should be conducted to explore this association further.

**Electronic supplementary material:**

The online version of this article (10.1186/s12885-018-5014-1) contains supplementary material, which is available to authorized users.

## Background

Bladder cancer, also known as urothelial cancer of the bladder, is the most common malignancy affecting the urinary system [1–3]. Treatment of bladder cancer has not advanced in the past 30 years [1]. The disease has a multifactorial etiology that includes environmental factors such as cigarette smoking, arsenic exposure and occupational exposure as well as genetic factors [4–6]. Genetic factors are the one of the most important factors associated with the onset of bladder cancer [7]. Smoking is a major risk factor for the development of this cancer, but the functional consequences of the carcinogens in tobacco smoke in terms of bladder cancer–associated metabolic changes remain poorly defined. Current evidence indicates that some gene polymorphisms are associated with bladder cancer morbidity [8–12].

Glutathione S-transferases (GSTs) play an important role in detoxification of various toxic compounds, such as carcinogens, and are a family of enzymes that include the glutathione S-transferase μ1 (GSTM1), θ1 (GSTT1) and π1 (GSTP1) classes, etc. [13]. They are important phase II detoxifying enzymes that catalyze the conjugation of reduced glutathione (GSH) to hydrophobic, electrophilic xenobiotic substances [14]. Genetic risk to malignant tumors has led to the accumulating attention to the investigations of genes polymorphism involved in process of carcinogenesis [15]. The gene polymorphisms of GSTs might influence the detoxification activities of the enzymes, predisposing individuals to cancers, such as oral squamous-cell carcinoma, gynecological cancer, breast cancer, prostate cancer, hepatocellular carcinoma, and colorectal cancer [16–21].

In the past few decades, most of the epidemiological investigations have focused on the relationship between the null genotypes for GSTM1-GSTT1 and bladder cancer susceptibility. However, available evidence is inadequate due to the sparseness of data or disagreements among reported studies. We performed this meta-analysis to investigate whether the dual-null GSTM1-GSTT1 genotype was associated with bladder cancer susceptibility.

## Methods

### Search strategy

We retrieved relevant published articles from the electronic databases of PubMed, Embase, the Cochrane Library and the China Biological Medicine Database (CBM-disc) on July 1, 2017, and we recruited eligible original articles for our meta-analysis. Key search terms consisted of [“glutathione S-transferases” OR “GSTs” OR “GSTM1” OR “GSTT1”] and [“bladder cancers” OR “bladder cancer”]. We identified additional articles through references cited in retrieved articles, and we also examined citations of retrieved articles and the previous meta-analyses.

### Inclusion and exclusion criteria

#### Inclusion criteria

(1) The endpoint of each study had to be bladder cancers. (2) The study had to include 2 comparison groups (bladder cancers vs. controls). (3) The study had to provide detailed data on genotype distribution.

#### Exclusion criteria

(1) Case reports, review articles and editorials. (1) Preliminary results not focused on GSTM1, GSTT1 or outcome. (3) Investigating the relationship of GST gene expression to disease. (4) Multiple publications.

### Quality appraisal

To evaluate the quality of the recruited articles that met the above-listed inclusion criteria, we used a quality score based on 7 aspects of genetic-association studies (Additional file 1: Table S1). Thakkinstian et al. [22] created the quality score form in 2005; its range spans from 0 (worst quality) to 12 (best quality). Two researchers who were responsible for literature retrieval appraised quality independent of one another, and a discussion was made until every respect was entirely consistent by comparison.

### Data extraction and synthesis

Two investigators independently excerpted the following information from each eligible study: first author’s surname, year of publication, and number of cases and controls for both the GSTM1 and GSTT1 genotypes. We calculated frequencies for both the disease group and the control group from the corresponding genotype distribution. Finally, we compared the results and resolved any disagreements by discussion. We tested the consistency of the data extracted by the 2 researchers, and any disagreement was again resolved by discussion.

### Statistical analysis

We performed all statistical analyses using Cochrane Review Manager Software, version 5 (RevMan 5; Cochrane Library, UK). We used *I*^*2*^ to test heterogeneity among the included studies, and we counted the pooled statistic using a fixed-effects model (Cochran–Mantel–Haenszel method), but switched to a random-effects model (DerSimonian–Laird method) when the *P*-value of the heterogeneity test was < 0.1. Results were expressed with odds ratios (ORs) for dichotomous data, and we also calculated 95% confidence intervals (CIs). *P* < 0.05 was required for the pooled OR to be statistically significant. We graphically judged publication bias from the Begg adjusted-rank correlation test [23] and the Egger regression asymmetry test [24] using the Stata version 12.0 (Stata Corporation, College Station, TX), and *P*-values < 0.1 were considered significant.

## Results

### Study characteristics for the GSTM1-null genotype and bladder cancer risk

**Fig. 1:**
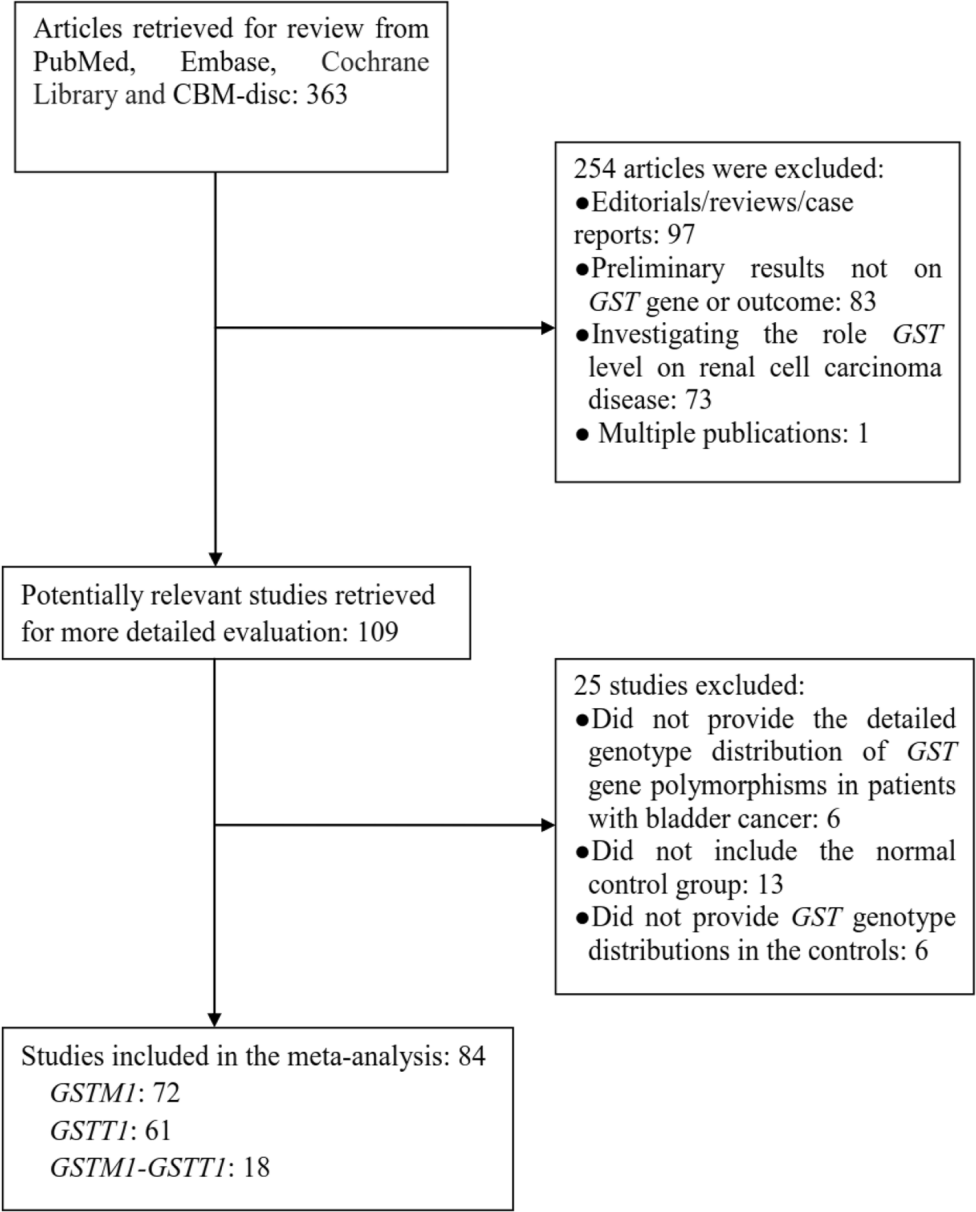
Flow chart of the study search and selection

We included 72 studies [25–96], which contained 20,239 case series and 24,393 controls, in our assessment of the relationship between the GSTM1-null genotype and bladder cancer susceptibility (Fig. 1 and Table 1). We extracted data of interest: first author’s surname, year of publication and number of cases and controls for the GSTM1-null genotype (Table 1). Average distribution frequency of the GSTM1-null genotype was 56.15% in the bladder cancer group and 46.97% in the control group, indicating that the GSTM1-null genotype was higher in the bladder cancer cases than in the controls (case/control = 1.20).

**Table 1:** Characteristics of the studies evaluating effects of the GSTM1-null genotypes on bladder carcinogen risk

Author, year	Country	Ethnicity	Source of controls	Quality Score	Case			Control		
					–	+	total	–	+	total
Bell 1993	USA	Overall	Population-based	9	111	89	200	85	115	200
		Caucasian	Population-based		61	39	100	50	50	100
		African	Population-based		50	50	100	35	65	100
Daly 1993	UK	Caucasian	Population-based	4	45	8	53	31	27	58
Zhong 1993	UK	Caucasian	Hospital-based	4	39	58	97	94	131	225
Lin 1994	USA, etc	Overall	Population-based	6	61	46	107	442	473	915
		Caucasian	Population-based		52	37	89	236	243	479
		Asian	Population-based		5	1	6	179	170	349
		African	Population-based		4	8	12	27	60	87
Okkels 1996	Denmark	Caucasian	Hospital-based	7	133	100	233	100	100	200
Anwar 1996	Egypt	African	Population-based	9	19	3	22	10	11	21
Brockmoller 1996	Germany	Caucasian	Hospital-based	8	217	157	374	192	181	373
Lafuente 1996	Egypt	African	Population-based	5	39	27	66	28	27	55
Katoh 1998	Japan	Asian	Hospital-based	9	66	46	112	50	62	112
Abdel-Rahman 1998	Egypt	African	Hospital-based	8	26	11	37	15	19	34
Salagovic 1999	Slovakia	Caucasian	Hospital-based	6	40	36	76	123	125	248
Mungan 2000	Netherlands	Caucasian	Hospital-based	4	38	23	61	30	39	69
Peluso 2000	Italy	Caucasian	Hospital-based	5	61	69	130	29	25	54
Schnakenberg 2000	Germany	Caucasian	Population-based	6	93	64	157	129	94	223
Steinhoff 2000	Germany	Caucasian	Hospital-based	7	80	55	135	57	70	127
Georgiou 2000	Greece	Caucasian	Hospital-based	6	56	33	89	56	91	147
Kim 2000	Korea	Asian	Hospital-based	6	78	34	112	128	97	225
Toruner 2001	Turkey	Asian	Hospital-based	8	75	46	121	55	66	121
Aktas 2001	Turkey	Asian	Population-based	4	56	47	103	70	132	202
Giannakopoulos 2002	Greece	Caucasian	Hospital-based	6	56	33	89	56	91	147
Kim 2002	Korea	Asian	Population-based	8	138	78	216	265	184	449
Lee 2002	Korea	Asian	Hospital-based	8	149	83	232	86	79	165
Ma 2002	China	Asian	Population-based	8	180	137	317	99	83	182
Schroeder 2003	USA	Mix	Hospital-based	8	137	93	230	101	112	213
Jong 2003	Korea	Asian	Population-based	9	75	51	126	99	105	204
Moore 2004	USA	Mix	Population-based	8	54	52	106	49	60	109
Srivastava 2004	India	Asian	Hospital-based	7	42	64	106	54	128	182
Hung 2004	France	Caucasian	Hospital-based	7	132	69	201	112	102	214
Saad 2005	UK	Caucasian	Population-based	8	45	27	72	40	41	81
Srivastava 2005	India	Asian	Population-based	10	140	230	370	43	63	106
Sobti 2005	India	Asian	Population-based	9	37	63	100	24	52	76
Garcia-Closas 2005	Spain	Caucasian	Hospital-based	9	716	422	1138	571	561	1132
Karagas 2005	USA	Mix	Population-based	9	210	134	344	309	233	542
Kim 2005	Korea	Asian	Hospital-based	7	92	61	153	73	80	153
McGrath 2006	USA	Mix	Population-based	11	109	82	191	483	439	922
Ouerhani 2006	Tunisia	African	Population-based	6	39	23	62	36	43	79
Murta-Nascimento 2007	Spain	Caucasian	Hospital-based	8	428	251	679	367	368	735
Moore 2007	Spain	Caucasian	Hospital-based	7	683	394	1077	524	498	1022
Cengiz 2007	Turkey	Caucasian	Hospital-based	6	34	17	51	22	31	53
Kellen 2007	Belgium	Caucasian	Population-based	8	312	267	579	597	466	1063
Zhao 2007	USA	Caucasian	Hospital-based	8	324	298	622	317	316	633
Shao 2008	China	Asian	Hospital-based	10	85	117	202	81	191	272
Yuan 2008	USA	Mix	Population-based	11	387	275	662	335	351	686
Covolo 2008	Italy	Caucasian	Hospital-based	7	128	69	197	111	100	211
Golka 2008	Germany	Caucasian	Hospital-based	7	184	109	293	88	88	176
Song 2009	China	Asian	Hospital-based	11	131	77	208	108	104	212
Altayli 2009	Turkey	Caucasian	Hospital-based	7	58	77	135	65	63	128
Grando 2009	Brazil	Mix	Population-based	7	40	60	100	33	67	100
Lin 2009	USA	Mix	Population-based	9	312	292	604	286	324	610
Zupa 2009	Italy	Caucasian	Population-based	8	13	10	23	68	53	121
Abd 2010	Egypt	African	Hospital-based	6	11	9	20	9	11	20
Moore 2011	USA	Mix	Hospital-based	10	653	400	1053	690	545	1235
Öztürk 2011	Turkey	Caucasian	Population-based	8	98	78	176	51	46	97
Rouissi 2011	Tunisia	African	Population-based	7	63	62	125	56	69	125
Salinas-Sonchez 2011	Spain	Caucasian	Hospital-based	5	109	92	201	78	115	193
Goerlitz 2011	Egypt	African	Hospital-based	9	344	274	618	332	289	621
Marenne 2012	Spain	Caucasian	Hospital-based	7	488	285	773	402	357	759
Ovsiannikov 2012	Germany	Caucasian	Hospital-based	6	102	94	196	123	112	235
Schwender 2012	Germany	Caucasian	Hospital-based	7	909	663	1572	863	876	1739
Henriquez-Hernondez 2012	Spain	Caucasian	Hospital-based	8	23	67	90	17	64	81
Lesseur 2012	New Hampshire	Caucasian	Hospital-based	9	378	275	653	508	420	928
Zhang 2012	USA	Mix	Hospital-based	10	381	329	710	402	380	782
Matic 2013	Serbia	Caucasian	Hospital-based	8	111	90	201	61	61	122
Savic-Radojevic 2013	Serbia	Caucasian	Hospital-based	6	45	35	80	32	28	60
Safarinejad 2013	Iran	Asian	Hospital-based	10	50	116	166	93	239	332
Wang 2013	China	Asian	Hospital-based	7	699	351	1050	834	570	1404
Berber 2013	Turkey	Caucasian	Hospital-based	7	54	60	114	51	63	114
Kang 2013	Korea	Asian	Hospital-based	9	65	45	110	103	117	220
Reszka 2014	Poland	Caucasian	Population-based	9	149	95	244	165	200	365
Ceylan 2015	Turkey	Caucasian	Hospital-based	8	22	43	65	31	39	70
Elhawary 2017	Saudi Arabia	Asian	Hospital-based	7	24	28	52	40	64	104
Ali 2017	Pakistan	Asian	Population-based	11	83	117	200	57	143	200

In the subgroup of patients and controls who smoked cigarettes, we included 24 studies [25, 30, 34, 35, 42, 43, 47, 48, 50, 51, 54–56, 64, 65, 68, 69, 73, 76, 83, 85, 91, 92, 95] (data not shown) containing 3724 case series and 3160 controls. Average distribution frequency of the GSTM1-null genotype was 55.67% in the bladder cancer group and 47.57% in the control group, indicating that the GSTM1-null genotype was significantly higher in the bladder cancer cases compared with the controls (case/control = 1.17).

### Study characteristics for GSTT1-null genotype and bladder cancer risk

We included 61 studies [29, 32–34, 36–40, 42–45, 47–53, 55–61, 63–69, 72–74, 76, 77, 79, 83–86, 89, 91, 92, 95–108] containing 13,041 case series and 16,739 controls in our assessment of the relationship between the GSTT1-null genotype and bladder cancer risk (Fig. 1 and Table 2). Average distribution frequency of the GSTT1-null genotype was 29.58% in the bladder cancer group and 26.67% in the control group, indicating that the GSTT1 -null genotype was higher in the bladder cancer cases compared with the controls (case/control = 1.11).

**Table 2:** Characteristics of the studies evaluating effects of the GSTT1-null genotype of on bladder carcinogen risk

Author, Year	Country	Ethnicity	Source of controls	Quality score	Case			Control		
					–	+	total	–	+	total
Brockmoller 1996	Germany	Caucasian	Hospital-based	8	66	308	374	78	295	373
Kempkes 1996	Germany	Caucasian	Population-based	7	20	93	113	31	139	170
Abdel-Rahman 1998	Egypt	African	Hospital-based	8	17	20	37	5	29	34
Katoh 1998	Japan	Caucasian	Hospital-based	9	46	66	112	59	53	112
Kim 1998	Korea	Asian	Hospital-based	7	18	49	67	29	38	67
Lee 1999	Korea	Asian	Hospital-based	7	93	65	158	66	65	131
Salagovic 1999	Slovakia	Caucasian	Hospital-based	6	21	55	76	42	206	248
Georgiou 2000	Greece	Caucasian	Hospital-based	6	5	84	89	16	131	147
Peluso 2000	Italy	Caucasian	Hospital-based	5	14	108	122	6	48	54
Kim 2000	Korea	Asian	Hospital-based	6	47	65	112	101	119	220
Steinhoff 2000	Germany	Caucasian	Hospital-based	7	20	115	135	17	110	127
Schnakenberg 2000	Germany	Asian	Hospital-based	6	28	129	157	48	175	223
Toruner 2001	Turkey	Asian	Hospital-based	8	24	97	121	21	100	121
Giannakopoulos 2002	Greece	Caucasian	Hospital-based	6	5	84	89	16	131	147
Lee 2002	Korea	Asian	Hospital-based	8	135	97	232	85	80	165
Ma 2002	China	Asian	Population-based	8	29	32	61	88	94	182
Kim 2002	Korea	Asian	Population-based	8	91	125	216	228	221	449
Gago-Dominguez 2003	USA	Mix	Population-based	8	50	146	196	34	142	176
Jong 2003	Korea	Asian	Hospital-based	9	68	58	126	113	91	204
Chen 2004	China	Asian	Population-based	8	32	30	62	51	30	81
Moore 2004	USA	Mix	Population-based	8	17	89	106	12	97	109
Hung 2004	France	Caucasian	Hospital-based	7	43	158	201	33	181	214
Srivastava 2004	India	Asian	Hospital-based	7	28	78	106	29	153	182
Sanyal 2004	Sweden	Caucasian	Population-based	8	66	204	270	12	110	122
Broberg 2005	Sweden	Caucasian	Population-based	9	7	54	61	22	132	154
Garcia-Closas 2005	Spain	Caucasian	Hospital-based	9	230	899	1129	248	873	1121
Saad 2005	UK	Caucasian	Population-based	8	26	46	72	14	67	81
Karagas 2005	USA	Mix	Population-based	9	53	83	136	301	458	759
Golka 2005	Dortmund	Caucasian	Hospital-based	8	30	106	136	38	125	163
Kim 2005	Korea	Asian	Hospital-based	7	71	82	153	89	64	153
Srivastava 2005	India	Asian	Population-based	10	28	78	106	79	291	370
Shao 2005	China	Asian	Population-based	7	204	201	405	195	194	389
Sobti 2005	India	Asian	Population-based	9	30	70	100	11	65	76
McGrath 2006	USA	Mix	Population-based	11	35	156	191	148	776	924
Ouerhani 2006	Tunisia	African	Population-based	6	26	36	62	35	44	79
Kogevinas 2006	Spain	Caucasian	Hospital-based	8	24	75	99	17	74	91
Cengiz 2007	Turkey	Caucasian	Hospital-based	6	18	33	51	11	42	53
Kellen 2007	Belgium	Caucasian	Population-based	8	30	164	194	61	319	380
Zhao 2007	USA	Caucasian	Hospital-based	8	103	520	623	115	519	634
Covolo 2008	Italy	Caucasian	Hospital-based	7	42	155	197	33	178	211
Yuan 2008	USA	Mix	Population-based	11	140	518	658	124	556	680
Song 2008	China	Asian	Hospital-based	7	71	37	108	58	54	112
Altayli 2009	Turkey	Caucasian	Hospital-based	7	31	104	135	9	119	128
Grando 2009	Brazil	Mix	Population-based	7	51	49	100	37	63	100
Song 2009	China	Asian	Hospital-based	11	110	98	208	105	107	212
Cantor 2010	Spain	Caucasian	Hospital-based	9	136	542	678	160	550	710
Moore 2011	USA	Mix	Hospital-based	10	210	794	1004	237	942	1179
Rouissi 2011	Tunisia	African	Population-based	7	30	95	125	38	87	125
Goerlitz 2011	Egypt	African	Hospital-based	9	147	470	617	156	464	620
Salinas-Sánchez 2011	Spain	Caucasian	Hospital-based	5	42	148	190	25	138	163
Lesseur 2012	New Hampshire	Caucasian	Hospital-based	9	106	556	662	143	780	923
Ovsiannikov 2012	Germany	Caucasian	Hospital-based	6	33	163	196	47	188	235
Henriquez-Hernondez 2012	Spain	Caucasian	Hospital-based	8	60	30	90	40	41	81
Berber 2013	Turkey	Caucasian	Hospital-based	7	31	83	114	16	98	114
Matic 2013	Serbia	Caucasian	Hospital-based	8	56	145	201	34	88	122
Safarinejad 2013	Iran	Asian	Hospital-based	10	35	131	166	69	263	332
Kang 2013	Korea	Asian	Hospital-based	9	64	46	110	128	92	220
Reszka 2014	Poland	Caucasian	Population-based	9	30	212	242	77	288	365
Ceylan 2015	Turkey	Caucasian	Hospital-based	8	19	46	65	9	61	70
Ali 2017	Pakistan	Asian	Population-based	11	34	166	200	26	174	200
Elhawary 2017	Saudi Arabia	Asian	Hospital-based	7	6	46	52	8	96	104

In the subgroup of patients and controls who smoked cigarettes, we included 21 studies [34, 42, 43, 47, 48, 50, 51, 55, 56, 64, 65, 68, 69, 73, 76, 83, 85, 91, 92, 95, 97] (data not shown) containing 3170 case series and 2793 controls. Average distribution frequency of the GSTT1-null genotype was 29.29% in the bladder cancer group and 28.65% in the control group-that is, similar in both groups (case/control = 1.02).

### Study characteristics for the dual-null GSTM1-GSTT1 genotype and bladder cancer risk

We included 18 studies [32, 37, 39, 43, 47, 48, 52, 55, 58, 60, 63, 65, 67, 79, 84, 85, 89, 96] containing 2426 case series and 3874 controls in our assessment of the relationship between the dual-null GSTM1-GSTT1 genotype and bladder cancer risk (Fig. 1 and Table 3). Average distribution frequency of the dual-null GSTM1-GSTT1 genotype was 16.78% in the bladder cancer group and 11.45% in the control group. Therefore, the dual-null GSTM1-GSTT1 genotype was significantly higher in the bladder cancer cases compared with the controls (case/control = 1.47).

**Table 3:** Characteristics of the studies evaluating effects of the GSTM1-GSTT1 dual-null genotype on bladder carcinogen risk

Author, Year	Country	Ethnicity	Source of controls	Quality score	Case			Control		
					null-null	non-null-null	total	null-null	non-null-null	total
Abdel-Rahman 1998	Egypt	African	Hospital-based	8	14	23	37	3	31	34
Steinhoff 2000	Germany	Caucasian	Hospital-based	7	12	123	135	4	123	127
Schnakenberg 2000	Germany	Caucasian	Population-based	6	12	145	157	31	192	223
Ma 2002	China	Asian	Population-based	8	16	45	61	54	128	182
Lee 2002	Korea	Asian	Hospital-based	8	83	149	232	37	128	165
Srivastava 2004	India	Asian	Hospital-based	7	16	90	106	9	173	182
Moore 2004	USA	Mix	Population-based	8	9	97	106	6	103	109
Hung 2004	France	Caucasian	Hospital-based	7	28	173	201	19	195	214
Srivastava 2005	India	Asian	Population-based	10	17	89	106	32	338	370
McGrath 2006	USA	Mix	Population-based	11	18	173	191	78	844	922
Song 2009	China	Asian	Hospital-based	11	77	131	208	50	162	212
Salinas-Sonchez 2011	Spain	Caucasian	Hospital-based	5	20	131	151	6	88	94
Ovsiannikov 2012	Germany	Caucasian	Hospital-based	6	17	179	196	29	206	235
Henriquez-Hernondez 2012	Spain	Caucasian	Hospital-based	8	17	73	90	8	73	81
Berber 2013	Turkey	Caucasian	Hospital-based	7	11	103	114	7	107	114
Safarinejad 2013	Iran	Asian	Hospital-based	10	38	128	166	73	259	332
Ceylan 2015	Turkey	Caucasian	Hospital-based	8	8	57	65	8	62	70
Elhawary 2017	Saudi Arabia	Asian	Hospital-based	7	0	104	104	0	208	208

### Association of the GSTM1-null genotype with bladder cancer risk

**Fig. 2:**
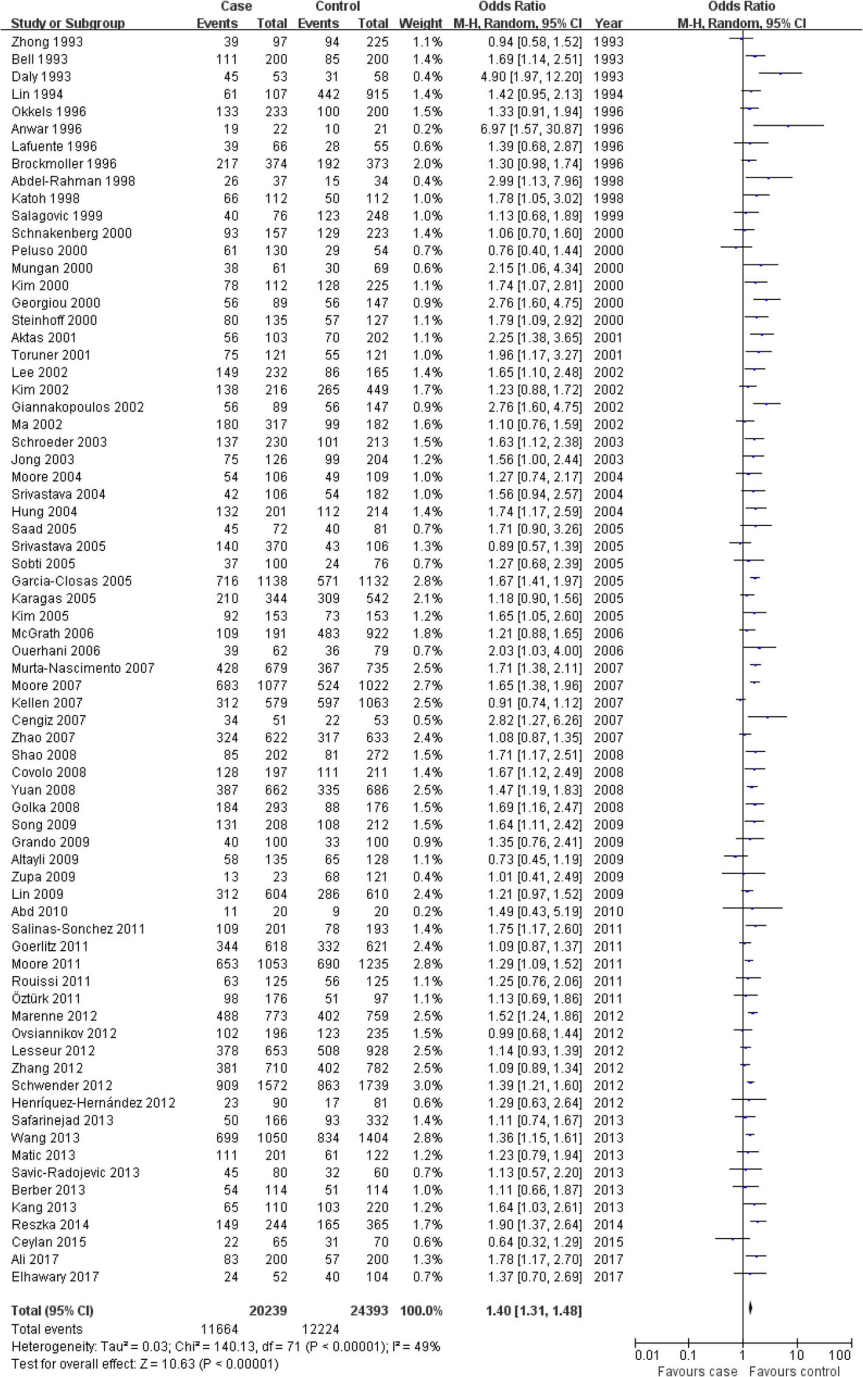
Association between the GSTM1-null genotype and bladder cancer susceptibility in the overall population

In this meta-analysis, we found that the GSTM1-null genotype was associated with bladder cancer risk in the overall population, and individually in whites, Africans and Asians (overall population: OR = 1.40, 95% CI: 1.31–1.48, *P*<0.00001; whites: OR = 1.39, 95% CI: 1.26–1.54, *P*<0.00001; Africans: OR = 1.54, 95% CI: 1.16–2.05, *P* = 0.003; Asians: OR = 1.45, 95% CI: 1.33–1.59, *P*<0.00001); as well as in controls from both hospital-based and population-based studies that included both high- and low-quality studies (Fig. 2 for the overall population; Table 4). In the meta-analysis for all patients and controls who smoked cigarettes, we found that the GSTM1-null genotype was associated with bladder cancer risk in the overall population, Asians and controls from both hospital-based and population-based studies that included both high- and low-quality studies. However, we did not find this relationship in whites or Africans (Table 4).

**Table 4:** Meta-analysis of the association of null genotypes of GSTM1, GSTT1 and dual-null genotype of GSTM1/GSTT1 with bladder carcinogens risk

Genetic contrasts	Group and subgroups	Studies Number	Q test *P* value	Model selected	OR (95% CI)	*P*
GSTM1						
- vs +	Overall	72	<0.00001	Random	1.40 (1.31,1.48)	<0.00001
	Caucasian	37	<0.00001	Random	1.39 (1.26,1.54)	<0.00001
	Asian	20	0.39	Fixed	1.45 (1.33,1.59)	<0.00001
	African	9	0.10	Random	1.54 (1.16,2.05)	0.003
	Hospital-based	46	0.0001	Random	1.42 (1.32,1.52)	<0.00001
	Population-based	26	0.003	Random	1.36 (1.21,1.53)	<0.00001
	High quality	54	0.0002	Random	1.37 (1.28,1.45)	<0.00001
	Low quality	18	0.0009	Random	1.58 (1.29,1.94)	<0.0001
GSTM1 (smoking)						
- vs +	Overall	24	0.02	Random	1.37 (1.19,1.59)	<0.0001
	Caucasian	10	0.007	Random	1.17 (0.85,1.59)	0.33
	Asian	7	0.63	Fixed	1.67 (1.32,2.11)	<0.0001
	African	3	0.22	Fixed	1.44 (0.95,2.17)	0.08
	High quality	17	0.02	Random	1.35 (1.14,1.60)	0.0005
	Low quality	7	0.27	Fixed	1.48 (1.12,1.96)	0.006
GSTT1						
- vs +	Overall	61	<0.00001	Random	1.11 (1.01,1.22)	0.03
	Caucasian	29	<0.00001	Random	1.16 (0.99,1.36)	0.07
	Asian	21	0.01	Random	1.05 (0.91,1.22)	0.51
	African	4	0.03	Random	1.07 (0.65,1.76)	0.79
	Hospital-based	40	<0.0001	Random	1.11 (0.99,1.24)	0.07
	Population-based	21	0.0002	Random	1.12 (0.94,1.35)	0.20
	High quality	52	<0.00001	Random	1.14 (1.03,1.26)	0.01
	Low quality	9	0.23	Fixed	0.93 (0.75,1.14)	0.49
GSTT1 (smoking)						
	Overall	21	0.67	Fixed	1.06 (0.93,1.20)	0.38
	Caucasian	9	0.84	Fixed	1.14 (0.91,1.43)	0.24
	Asian	7	0.62	Fixed	1.00 (0.77,1.30)	0.99
	African	2	0.41	Fixed	0.60 (0.36,1.02)	0.06
	High quality	16	0.52	Fixed	1.06 (0.93,1.22)	0.37
	Low quality	5	0.64	Fixed	1.01 (0.70,1.48)	0.94
Dual-null genotype of GSTM1/GSTT1						
	Overall	18	0.003	Random	1.48 (1.15,1.92)	0.002
	Caucasian	8	0.03	Random	1.30 (0.83,2.03)	0.25
	Asian	7	0.04	Random	1.62 (1.15,2.28)	0.006
	Hospital-based	13	0.03	Random	1.71 (1.28,2.28)	0.0003
	Population-based	5	0.06	Random	1.07 (0.67,1.71)	0.77
	High quality	15	0.11	Fixed	1.61 (1.36,1.91)	<0.00001
	Low quality	3	0.04	Random	0.86 (0.40,1.85)	0.70

### Association of the GSTT1-null genotype with bladder cancer risk

**Fig. 3:**
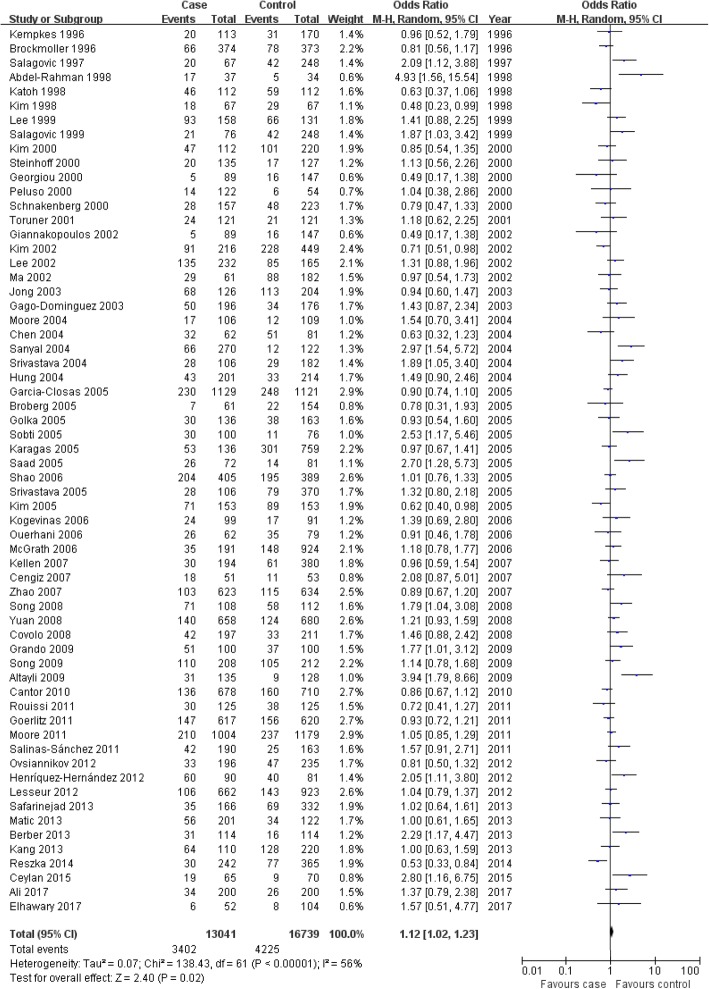
Association between the GSTT1-null genotype and bladder cancer susceptibility in the overall population

In this study, we found that the GSTT1-null genotype was associated with bladder cancer risk in the overall population, and controls from hospital-based studies that included high-quality studies; but not with bladder cancer risk in whites, Africans, Asians or controls from population-based studies that included low-quality studies (overall population: OR = 1.11, 95% CI: 1.01–1.22, *P* = 0.03; whites: OR = 1.16, 95% CI: 0.99–1.36, *P* = 0.07; Africans: OR = 1.07, 95% CI: 0.65–1.76, *P* = 0.79; Asians: OR = 1.05, 95% CI: 0.91–1.22, *P* = 0.51; Fig. 3 for overall population; Table 4). However, in controls from either hospital-based or population-based studies that included both high- and low-quality studies, or in the meta-analysis for all patients and controls who smoked cigarettes, we found that the GSTT1-null genotype was not associated with bladder cancer risk in the overall population, or in individual white, African or Asian populations (Table 4).

### Association of dual-null GSTM1-GSTT1 genotype with bladder cancer risk

**Fig. 4:**
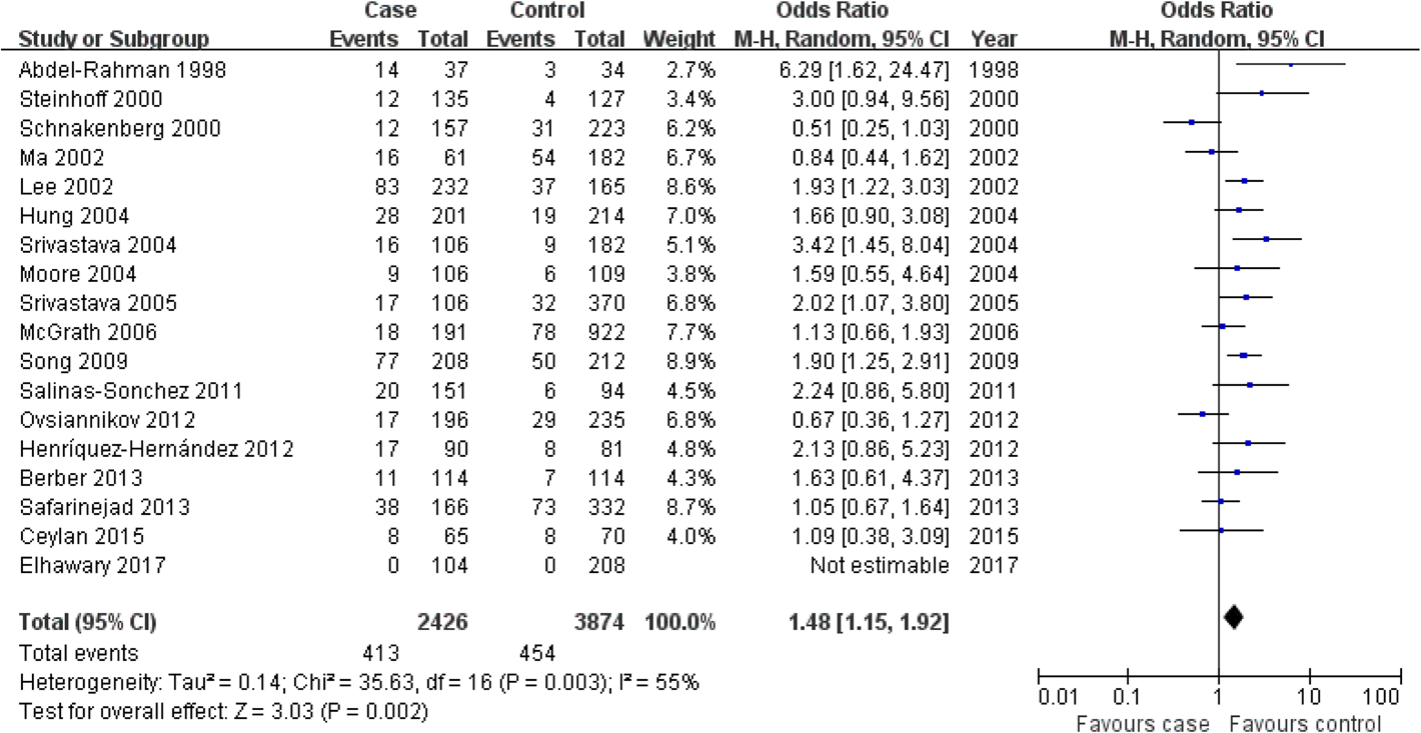
Association between the dual-null GSTM1–GSTT1 genotype and bladder cancer risk in the overall population

We found an association between the dual-null GSTM1-GSTT1 genotype and bladder cancer risk in the overall population, Asians and controls from hospital-based studies that included high-quality studies (overall population: OR = 1.48, 95% CI: 1.15–1.92, *P* = 0.002; Asians: OR = 1.62, 95% CI: 1.15–2.28, *P* = 0.006; Fig. 4 for overall population; Table 4). However, the dual-null GSTM1-GSTT1 genotype was not associated with onset of bladder cancer in whites or in controls from population-based studies that included low-quality studies (whites: OR = 1.30, 95% CI: 0.83–2.03, *P* = 0.25; Table 4).

### Evaluation of publication bias

**Fig. 5:**
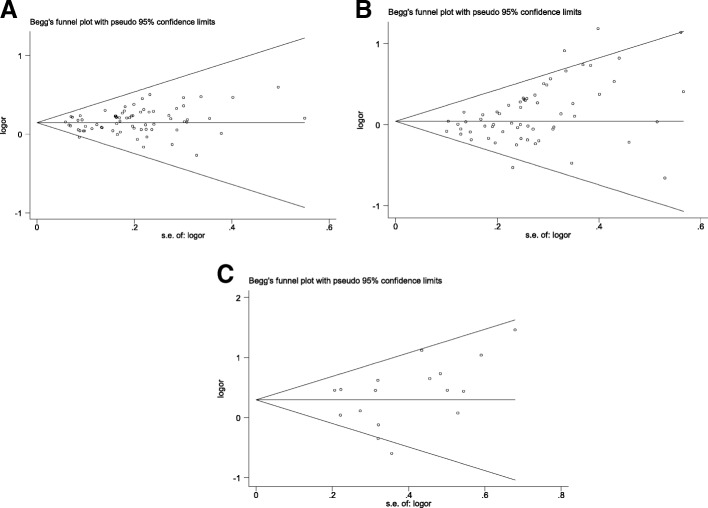
Publication bias. **a** GSTM1-null genotype. **b** GSTT1-null genotype. **c** Dual-null GSTM1–GSTT1 genotype

We performed a publication bias test for the association of the GSTM1-null, GSTT1-null and dual-null GSTM1-GSTT1 genotypes with bladder cancer risk in the overall population. There was no bias for the association of the dual-null GSTM1-GSTT1 genotype with bladder cancer risk, but there was for the GSTM1- and GSTT1-null genotypes (GSTM1-null: Begg *P* = 0.100, Egger *P* = 0.052; GSTT1-null: Begg *P* = 0.001, Egger *P* = 0.002; dual-null GSTM1–GSTT1: Begg *P* = 0.343, Egger *P* = 0.236; Fig. 5).

## Discussion

Research on single-nucleotide polymorphisms have focused mainly on their impact on tumor suppressor genes, metabolic-enzyme genes, and DNA repair genes, etc. Understanding disease susceptibility and pathogenesis and using them to guide diagnosis and individual treatment choice constitute an important new therapeutic approach [109]. In this study, we found that the average distribution frequency of the GSTM1-null genotype was significantly higher in bladder cancer cases than in controls (case/control = 1.20). In the subgroup of patients and controls who smoked cigarettes, it was also higher in the bladder cancer case group compared with the control group (case/control = 1.17). This might indicate that the GSTM1-null genotype was associated with bladder cancer risk in the overall population, including whites, Africans, Asians, and controls from both hospital-based and population-based studies that included both high- and low-quality studies. In the meta-analysis for all patients and controls who smoked cigarettes, we found that the GSTM1-null genotype was associated with bladder cancer risk in the overall population, Asians, and controls from both hospital-based and population-based studies that included both high- and low-quality studies. The sample size of our meta-analysis was larger than those of other meta-analyses [61, 110–112], and therefore our results might be more robust. However, our tests for publication bias, the GSTM1 studies were found to be positive. Therefore, the positive association between the GSTM1-null genotype and bladder cancer should be reassessed in the future.

The average distribution frequency of the GSTT1-null genotype was higher in the bladder cancer case group than in the control group (case/control = 1.11). In the subgroup of patients and controls who smoked cigarettes, it was similar in both groups (case/control = 1.02). This might tell us that the GSTT1-null genotype was associated with bladder cancer risk. For confirmation, we performed a meta-analysis, which further showed the GSTT1-null genotype to be associated with bladder cancer risk in the overall population, whites and controls from hospital-based studies that included high-quality studies. In the meta-analysis for all patients and controls who smoked cigarettes, we found that the GSTT1-null genotype was not associated with bladder cancer risk in the overall population, whites, Africans, Asians or controls from both hospital-based and population-based studies that included both high- and low-quality studies. Our results indicate that the GSTT1-null genotype does not predict the risk of bladder cancer. The sample size of our meta-analysis was larger than those of other meta-analyses [111, 112], suggesting that our conclusion might be more robust. However, publication bias was also found for GSTT1. Therefore, further studies are required.

Average distribution frequency of the dual-null GSTM1-GSTT1 genotype in the bladder cancer group was slightly higher than in the control group (case/control = 1.47), indicating a possible association between the dual-null GSTM1-GSTT1 genotype and bladder cancer risk. Meta-analysis further revealed an association between the dual-null GSTM1-GSTT1 genotype and bladder cancer risk in the overall population, Asians and controls from hospital-based studies that included high-quality studies. No publication bias was found for this meta-analysis, and the conclusion was robust.

In a previous study, García-Closas et al. [61] conducted a meta-analysis of 28 studies of GSTM1 and reported that the GSTM1-null genotype both increased the overall risk of bladder cancer and posed similar relative risks for both smokers and non-smokers. This finding suggested that GSTM1 lowers the risk of bladder cancer through mechanisms that are not specific to the detoxification of polycyclic aromatic hydrocarbons in tobacco smoke. Engel et al. [110] performed a meta-analysis of GSTM1 and bladder cancer that included 17 studies and reported that the GSTM1-null status is associated with a modest increase in the risk of bladder cancer, and that there was no evidence of multiplicative interaction between the GSTM1-null genotype and once and current smoking in relation to bladder cancer. A meta-analysis by Yu et al. [112] included 48 case–control studies for GSTM1*-null* and 57 studies for GSTT1, and suggested that the GSTM1- and GSTT1-null genotypes might both be related to higher bladder cancer risk. Yu et al. [111] also performed a meta-analysis to investigate the association between GSTM1-GSTT1 deletion polymorphisms and bladder cancer susceptibility, including 46 studies of GSTM1-null, 54 of GSTT1 and 10 of dual-null GSTM1-GSTT1. All 3 genotypes were associated with increased bladder cancer risk. In our meta-analysis, we included 72 studies for GSTM1-null, 62 for GSTT1-null and 18 for dual-null GSTM1-GSTT1 genotypes. These results from the meta-analyses mentioned above were similar to our results. However, the sample size of our meta-analysis was larger than the previous meta-analyses, and the results from our studies might be more robust. Furthermore, we initially conducted a meta-analysis that showed no evidence of multiplicative interaction between the GSTT1-null genotype and smoking in relation to bladder cancer.

Smoking is a known risk factor for bladder cancer [113], and the products of GSTs help detoxify the polycyclic aromatic hydrocarbons found in tobacco smoke [114]. Our study suggests that the GSTM1-null genotype might play a role in such detoxification, but the GSTT1-null genotype does not. However, more studies should be conducted to confirm this.

GSTM1-null, GSTT1-null and dual-null GSTM1-GSTT1 genotypes play an important role in detoxification of various toxic compounds, such as carcinogens. In this meta-analysis, it indicated that GSTM1-null, GSTT1-null and dual-null GSTM1-GSTT1 genotypes were risk factors to susceptibility of bladder cancer, and took part in the pathogenesis of bladder cancer.

There were limitations in our meta-analysis. First, age might be a source of heterogeneity, but it was difficult to stratify the different ages in the reports prior to pooling the results, for the reason that the ages from most of the included studies were different. So, no conclusions can be drawn regarding the impact of GSTs on age of onset. Furthermore, heterogeneity and publication bias were both significant for GSTM1-null and GSTT1-null. Subgroup analyses were performed to find out any effect modifier, but the reason was not clear.

## Conclusion

Our results supported that the GSTM1-null, GSTT1-null and dual-null GSTM1–GSTT1 genotypes might be associated with the onset of bladder cancers. However, more association investigations are required to further clarify these relationships.

## Additional file

Additional file 1: **Table S1.** Scale for Quality Assessment. (DOC 42 kb)
