# Supplementary material for: Association of Glutathione S-transferase gene polymorphism with bladder Cancer susceptibility
Source: BMC Cancer. 2018 Nov 12;18:1088. doi: 10.1186/s12885-018-5014-1 (PMC6233535; doi:10.1186/s12885-018-5014-1)
Supplement: Supplementary file 1 — Table S1. Scale for Quality Assessment. (DOC 42 kb) [file 12885_2018_5014_MOESM1_ESM.doc]

**Table S1：Scale for Quality Assessment**

| **Quality parameters** | **Score** |
| --- | --- |
| Representativeness of case |  |
| Selected from population cancer registry | 2 |
| Selected from hospital | 1 |
| No method of selection described | 0 |
| Representativeness of control |  |
| Population-based or healthy volunteers | 3 |
| Blood donors | 2 |
| Hospital-based (cancer-free patients) | 1 |
| Not described | 0 |
| Ascertainment of renal cell carcinoma |  |
| Histopathologic confirmation | 2 |
| Diagnosis of renal cell carcinoma by patient medical record or by patient history | 1 |
| Not described | 0 |
| Genotyping examination |  |
| The genotypes of all DNA samples were reassessed twice to confirm the results and ensure reproducibility | 1 |
| Undone or not mentioned | 0 |
| Sample size (total number of cases and controls) |  |
| Larger than 200 | 2 |
| Larger than 100, but less than 200 | 1 |
| Less than 100 | 0 |
| Matching of case and control participants |  |
| Controls matched with cases more than one variable (i.e., age, gender and ethnicity, cigarette smoking habits) | 2 |
| Controls matched with cases only one variable (i.e., age, gender or ethnicity) | 1 |
| Not matched or not descried | 0 |
| Total | 12 |

Studies were categorized as ‘‘high quality’’ if the quality score was ≥7; otherwise, studies were categorized as ‘‘low quality’’
